# Draft Genome Sequence of Adlercreutzia equolifaciens IPLA 37004, a Human Intestinal Strain That Does Not Produce Equol from Daidzein

**DOI:** 10.1128/MRA.01537-19

**Published:** 2020-02-20

**Authors:** Lucía Vázquez, Ana Belén Flórez, Baltasar Mayo

**Affiliations:** a Departamento de Microbiología y Bioquímica, Instituto de Productos Lácteos de Asturias (IPLA-CSIC), Villaviciosa, Asturias, Spain; b Instituto de Investigación Sanitaria del Principado de Asturias (ISPA), Oviedo, Asturias, Spain; University of Maryland School of Medicine

## Abstract

Equol is an intestinal bacterial metabolite derived from the isoflavone daidzein and has beneficial health effects. Most equol producers belong to the family *Coriobacteriaceae*, which includes species such as Adlercreutzia equolifaciens. Here, we report the draft genome sequence of A. equolifaciens IPLA 37004, a human isolate that does not produce equol.

## ANNOUNCEMENT

Among isoflavones and their metabolites, the bacterial daidzein-derived compound (*S*)-(−)-equol has the strongest estrogenic activity (1) and antioxidant action (2), by which it can positively influence human health (3). However, only 30 to 60% of humans harbor equol-producing microbes in their intestines. Most equol-producing bacteria belong to the family *Coriobacteriaceae* of the phylum *Actinobacteria*, which includes species such as Adlercreutzia equolifaciens, Asaccharobacter celatus, Enterorhabdus mucosicola, Slackia isoflavoniconvertens, and Slackia equolifaciens (3). However, whether equol production is a family-, species-, or strain-specific characteristic is not yet known.

Strain IPLA 37004 was isolated from fecal samples from a woman with an equol-producing microbiota, using agar plates of Gifu anaerobic medium (GAM) (Nissui) supplemented with 5 g liter^−1^ arginine (GAM-Arg; Merck) and cultivation under strict anaerobic conditions. Sequencing and sequence comparison of the 16S rRNA gene of IPLA 37004 identified the strain as A. equolifaciens. Surprisingly, the strain did not produce equol when cultured in GAM-Arg with isoflavones, daidzein, or dihydrodaidzein. To get insights regarding its non-equol-producing phenotype, the genome of A. equolifaciens IPLA 37004 was sequenced, and its sequence was analyzed.

After growth of the strain in GAM-Arg, total genomic DNA from IPLA 37004 was extracted and purified using the DNeasy blood and tissue kit (Qiagen), as suggested by the manufacturer. For genome sequencing, a library was constructed using SPRIworks fragment library system I (Beckman Coulter), according to the manufacturer’s instructions. Paired-end sequencing (2 × 150-bp reads) was performed at Eurofins Genomics on a HiSeq sequencer (Illumina) (sequence mode, NovaSeq 6000; S2 flow cell; paired-end 150-bp reads; Xp flowchart). The average genome coverage approached 100×, yielding a total of 5,568,276 bp of adapter-free and quality-filtered reads after the use of Trimmomatic (4) and FastQC (5) software packages. *De novo* assembly of quality-filtered nontrimmed reads in contigs was accomplished using SPAdes v3.6.2 (6), setting the parameters with a k value of 125 or 127 and only-assembler. Then, the NCBI Prokaryotic Genome Annotation Pipeline (7) was used to predict and annotate the open reading frames. Whole-genome comparisons were achieved by using the multiple genome alignment software package Mauve v2.3.1 (8). Finally, BLAST v2.10.0 searches (BLASTp suite, NCBI) were used to determine homology between proteins of different strains.

The genome of A. equolifaciens IPLA 37004 consisted of 2,664,741 bp in 169 contigs longer than 200 bp, with a G+C content of 63.7%. The maximum contig length was 325,998 bp, and the *N*_50_ and *N*_90_ values were 101,827 and 25,297 bp, respectively. A total of 2,310 genes were identified in the IPLA 37004 genome, including 2,251 coding sequences, of which 2,198 encoded complete proteins and 53 were identified as pseudogenes. In addition, 59 genes encoded RNA molecules, including 8 rRNAs, 48 tRNAs, and 3 noncoding RNAs. Genes encoding reductases homologous to those of A. equolifaciens DSM 19450^T^ involved in equol formation (9) were not detected in the IPLA 37004 genome. Whole-genome comparison of DSM 19450^T^ and IPLA 37004 identified shared flanking genes upstream and downstream of the equol locus in A. equolifaciens DSM 19450^T^. The deduced amino acids of the shared genes showed an identity range of 80 to 99%. The gene cluster of DSM 19450^T^ encompassed a region of about 11 kbp, which was absent in IPLA 37004.

### Data availability.

The genome sequence of A. equolifaciens IPLA 37004 was deposited in GenBank under accession number VJNE00000000. The fastq files of the raw reads were deposited in the NCBI SRA under accession number SRR9657807.
